# Maternal Exercise Mediates Hepatic Metabolic Programming via Activation of AMPK-PGC1α Axis in the Offspring of Obese Mothers

**DOI:** 10.3390/cells10051247

**Published:** 2021-05-19

**Authors:** Philipp Kasper, Saida Breuer, Thorben Hoffmann, Christina Vohlen, Ruth Janoschek, Lisa Schmitz, Sarah Appel, Gregor Fink, Christoph Hünseler, Alexander Quaas, Münevver Demir, Sonja Lang, Hans-Michael Steffen, Anna Martin, Christoph Schramm, Martin Bürger, Esther Mahabir, Tobias Goeser, Jörg Dötsch, Eva Hucklenbruch-Rother, Inga Bae-Gartz

**Affiliations:** 1 Clinic for Gastroenterology and Hepatology, Faculty of Medicine and University Hospital Cologne, University of Cologne, D-50937 Cologne, Germany; philipp.kasper@uk-koeln.de (P.K.); slang@ucsd.edu (S.L.); hans-michael.steffen@uk-koeln.de (H.-M.S.); anna.martin@uk-koeln.de (A.M.); christoph.schramm@uk-koeln.de (C.S.); martin.buerger@uk-koeln.de (M.B.); tobias.goeser@uk-koeln.de (T.G.); 2 Department of Pediatrics and Adolescent Medicine, Faculty of Medicine and University Hospital Cologne, University of Cologne, D-50937 Cologne, Germany; saida.breuer@uk-koeln.de (S.B.); thorben.hoffmann@uk-koeln.de (T.H.); christina.vohlen@uk-koeln.de (C.V.); ruth.janoschek@uk-koeln.de (R.J.); lisa.schmitz@uk-koeln.de (L.S.); sarah.appel@uk-koeln.de (S.A.); Gregor.fink@uk-koeln.de (G.F.); christoph.huenseler@uk-koeln.de (C.H.); joerg.doetsch@uk-koeln.de (J.D.); eva.rother@uni-koeln.de (E.H.-R.); 3 Department of Pathology, Faculty of Medicine and University Hospital Cologne, University of Cologne, D-50937 Cologne, Germany; alexander.quaas@uk-koeln.de; 4 Charité Campus Mitte and Campus Virchow Clinic, Department of Hepatology and Gastroenterology, Charité University Medicine Berlin, D-13353 Berlin, Germany; muenevver.demir@charite.de; 5 Department of Medicine, University of California San Diego, La Jolla, CA 92093, USA; 6 Comparative Medicine, Center for Molecular Medicine Cologne (CMMC), Faculty of Medicine and University Hospital Cologne, D-50937 Cologne, Germany; esther.mahabir-brenner@uni-koeln.de

**Keywords:** NAFLD, metabolic health, AMPK, gestational exercise, perinatal programming

## Abstract

Maternal obesity is associated with an increased risk of hepatic metabolic dysfunction for both mother and offspring and targeted interventions to address this growing metabolic disease burden are urgently needed. This study investigates whether maternal exercise (ME) could reverse the detrimental effects of hepatic metabolic dysfunction in obese dams and their offspring while focusing on the AMP-activated protein kinase (AMPK), representing a key regulator of hepatic metabolism. In a mouse model of maternal western-style-diet (WSD)-induced obesity, we established an exercise intervention of voluntary wheel-running before and during pregnancy and analyzed its effects on hepatic energy metabolism during developmental organ programming. ME prevented WSD-induced hepatic steatosis in obese dams by alterations of key hepatic metabolic processes, including activation of hepatic ß-oxidation and inhibition of lipogenesis following increased AMPK and peroxisome-proliferator-activated-receptor-γ-coactivator-1α (PGC-1α)-signaling. Offspring of exercised dams exhibited a comparable hepatic metabolic signature to their mothers with increased AMPK-PGC1α-activity and beneficial changes in hepatic lipid metabolism and were protected from WSD-induced adipose tissue accumulation and hepatic steatosis in later life. In conclusion, this study demonstrates that ME provides a promising strategy to improve the metabolic health of both obese mothers and their offspring and highlights AMPK as a potential metabolic target for therapeutic interventions.

## 1. Introduction

Paralleling the global epidemic of obesity, the prevalence of maternal overweight conditions and obesity has risen rapidly over the last decades and has become a growing health concern in industrialized countries, as it is associated with adverse long-term health consequences for both mothers and offspring [1,2]. In the United States, about 50–60% of women of reproductive age (20–39 years) are currently overweight and about 25–35% are obese, with a similar frequency distribution in Europe [1,3,4].

Obese pregnant women are at increased risk for gestational diabetes, hypertensive pregnancy disorders, and preterm birth [5,6]. Moreover, maternal overweight and obesity during pregnancy are closely linked to an adverse intrauterine environment and the development of metabolic diseases in the offspring. Offspring of obese women are at increased risk to become overweight or obese and are more prone to develop metabolic disorders, including type 2 diabetes, coronary heart disease, and non-alcoholic fatty liver disease (NAFLD) [7,8,9,10].

NAFLD is a metabolic disease of particular relevance since the liver mediates the maintenance of systemic metabolic homeostasis, and liver dysfunction can have deleterious effects on whole-body metabolic health [11,12]. NAFLD has become the most common cause of chronic liver disease worldwide and encompasses a spectrum of liver disorders ranging from simple hepatocellular steatosis (non-alcoholic fatty liver, NAFL) to inflammatory non-alcoholic steatohepatitis (NASH) with or without fibrosis [13,14]. The pathogenesis of NAFLD is multifactorial and characterized by an altered hepatic energy metabolism [15,16]. Due to its pathophysiological characteristics and its association with metabolic dysregulation, NAFLD is expected to be renamed to metabolic dysfunction-associated fatty liver disease (MAFLD) in the near future [17]. 

One key regulator of hepatic energy homeostasis is the adenosine monophosphate (AMP)-activated protein kinase (AMPK) [18,19]. AMPK is a ubiquitously expressed serine/threonine kinase complex and key sensor in maintaining cellular energy homeostasis. AMPK is activated in response to a variety of metabolic stressors, such as fasting or physical exercise, that change the cellular AMP to adenosine triphosphate (ATP) ratio by increasing ATP consumption or reducing ATP production [18,20,21,22]. Once activated, AMPK redirects the cellular metabolism toward increased catabolism and decreased anabolism via the phosphorylation of key metabolic substrates and transcriptional regulators (e.g. PGC-1α) to restore cellular energy homeostasis [18,22]. Since AMPK activation may protect against obesity, hepatic lipid accumulation, and obesity-induced insulin resistance, AMPK represents a promising target for both the prevention and treatment of metabolic diseases [22,23,24].

However, while accumulating evidence indicates that AMPK has immediate beneficial effects in those individuals in whom it is activated (e.g., by exercise), it remains largely unclear whether these protective effects can also be transferred to subsequent generations. 

The paradigm that offspring metabolic health is significantly shaped by the maternal metabolic phenotype and alterations in the early life environment is known as the ‘developmental origins of health and disease‘ hypothesis (or ‘fetal metabolic programming concept‘) and was first proposed by Barker and Halles [25,26,27]. Although studies have demonstrated that adverse maternal conditions (e.g., maternal obesity) impair offspring metabolic health and that preventive interventions, such as maternal exercise during critical periods of developmental programming, seem to be able to reduce the susceptibility to and severity of metabolic dysfunction, the underlying molecular mechanisms remain poorly understood.

Using a rodent model of maternal diet-induced obesity, we investigated whether a voluntary exercise intervention before and during pregnancy could improve maternal hepatic metabolic health via regulating AMPK-mediated mechanisms and explored to what extent potential programming effects affect hepatic metabolism of the offspring.

## 2. Materials and Methods

The present study was carried out by the Department of Pediatrics of the University Hospital of Cologne. The study was approved by the appropriate governmental authority (Institutional protocol number of the animal welfare application: AZ 81.02.04.2017.A442, Landesamt für Natur, Umwelt und Verbraucherschutz Nordrhein-Westfalen, Germany). All animal experiments were carried out in accordance with the German Animal Welfare Law. Animal care and use were performed by qualified individuals, supervised by a veterinarian and all standards regarding the work with animals were met. The manuscript complies with the Animals in Research: Reporting In Vivo Experiments (ARRIVE) guidelines [28].

### 2.1. Animal Model

**Figure 1:**
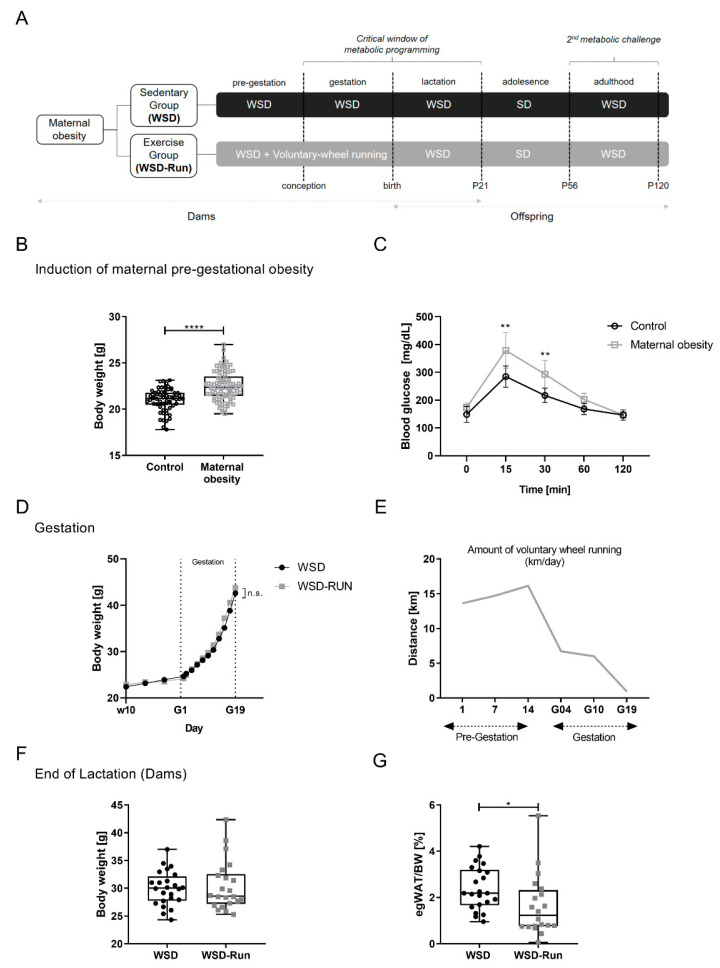
Maternal exercise before and during pregnancy alters the body composition of obese dams. (**A**) Experimental design: WSD, sedentary obese dams; WSD-Run, obese dams performing voluntary wheel running before and during pregnancy. (**B**) Induction of a model of maternal obesity. Female mice (n = 86) were fed an obesogenic western style diet for 8 weeks for induction of an obese phenotype and a cohort of obese dams, respectively. A separate cohort (n = 62) of female mice receiving a standard laboratory chow diet served as lean control group (Control). (**C**) Glucose tolerance was measured via intraperitoneal glucose tolerance test (ipGTT) at week 8–9 indicating altered glucose tolerance in obese dams (*n* = 10/group). Thus, before subsequent division into an ‘exercise’ and ‘sedentary’ group, the maternal obesity cohort exhibited a disturbed metabolic phenotype with WSD induced obesity and impaired glucose metabolism. (**D**) Weight gain during pregnancy. (**E**) Average running distance of the exercise group (WSD-Run) before and during pregnancy in km/day. (**F**) Total body weight of dams at the end of lactation at postnatal day (P) 21 (WSD group (n = 24), WSD-Run group (n = 22)). (**G**) Epigonadal fat pad weight at P21 (WSD group (n = 22), WSD-Run group (n = 20)). Data are presented as mean ± standard deviation; * *p* < 0.05, ** *p* < 0.01, **** *p* < 0.0001. BW, body weight; egWAT, epigonadal white adipose tissue; p, postnatal day; WSD, western-style diet; WSD-Run, western-style diet induced obese dams performing voluntary wheel running.

First, three-week-old female C57BL/6N mice, obtained from Charles River Laboratories (Germany), were fed an energy-rich obesogenic Western-style diet (#E15744-344 Ssniff, Germany; containing 387 g/kg carbohydrates, 236 g/kg protein, and 220 g/kg fat; total metabolizable energy (MetE) 4610 kcal/kg, 45% of MetE from fat, 20% of MetE from protein, 35% of MetE from carbohydrates of which 19% were sugar; see Supplementary Table S3) for 8 weeks during preconception to induce a maternal obesity cohort (n = 86). A separate cohort of n = 62 control mice were fed a standard laboratory chow diet (#.R/M-H SSniff, Germany; containing 412 g/kg carbohydrates, 190 g/kg protein, and 33 g/kg fat; total metabolizable energy 3220 kcal/kg, 9% of total MetE from fat) and served as the lean control group (Figure 1B). 

Obese dams from the maternal obesity cohort were then divided into either a voluntary wheel running exercise group (WSD-Run) or sedentary control group (WSD) at week 10. The running intervention group (WSD-Run) had unrestricted access to a voluntary running wheel (Supplementary Figure S2) for two weeks prior to conception and during pregnancy, which was equipped with a tachometer measuring distance (km), average speed (km/h) and time (h:m) as described before [29]. Dams of both groups (WSD and WSD-Run) were mated between week 12 and 13. Male breeders were fed with standard chow and had limited access to the running wheel during the mating process for 48 h. 

During gestation, the body weight of the dams was monitored every second day from gestational day (G) 1 to G19. Postpartum, the body weight of both offspring groups (WSD vs. WSD-Run, named after maternal conditions) was monitored every second day, starting immediately after birth at postnatal day 1 (P1) up to P21. Running wheels were removed following parturition in order to restrict exercise to the prenatal period and to ensure sufficient caretaking of the pups by the dam. The dams were maintained on WSD throughout the pregnancy and lactation periods. On P3, the litter size was randomly adjusted to six for each litter. At P21, dams and a part of the offspring were sacrificed by CO2-asphyxiation for organ harvest and non-fasted blood samples were collected via intracardial puncture for further analyses. Organs were weighed, immediately snap-frozen in liquid nitrogen, and stored at −80 °C for biochemical analyses. Liver tissue was additionally fixed in 4% paraformaldehyde for histological analyses. Post-weaning (P21), all of the remaining offspring were weaned on a standard chow diet for five weeks until P56 (Figure 1A). From P57 to P120, all offspring were again subjected to the obesogenic Western-style diet to induce a second metabolic challenge in later life. After nine weeks of WSD feeding, at P120, all animals were sacrificed via CO2-inhalation and processed as described before. A maximum of one pup per litter was studied at each time point to exclude litter-dependent bias.

For the duration of the whole study, all C57BL/6N mice were bred and held at the animal facility of the Department of Pharmacology of the University Hospital of Cologne (Cologne, Germany), where they had free access to the experimental diets and water ad libitum. Mice were housed in a room maintained at 22 ± 2 °C, exposed to humidity of 50% to 60% and a 12/12 h light/dark cycle. All breeding colonies were kept in individually ventilated cages (IVCs, Blue Line Cages type II long, Tecniplast, Italy). To exclude gender influences, all studies were performed using male offspring in accordance with previous study settings [30,31,32]. Cumulative food intake was measured in dams at week 10. The experimental protocol is shown schematically in Figure 1A.

As the present study primarily aimed to analyze whether maternal exercise during an obese pregnancy effectively reduced detrimental metabolic effects, we were not seeking to investigate the impact of maternal exercise during a lean pregnancy. Therefore, we did not include an additional exercised control group of lean dams and their offspring to ensure that we were complying with the ARRIVE guidelines, and only utilized animals required to achieve our specific research aims.

### 2.2. Analytical Procedures and Biochemical Measurements

Blood samples were taken when animals were sacrificed via intracardial puncture at P21. Blood samples were stored at room temperature for 30 min after extraction and centrifuged for 10 min at 3000× *g* and 4 °C, then stored at −20 °C until analyzed.

### 2.3. Intraperitoneal Glucose Tolerance Test (ipGTT) and Insulin Tolerance Test (ipITT) 

In dams, ipGTT and ipITT were performed at weeks 8–9. For both tests, animals were fasted for 6 h during light phase (07.00 a.m. to 01.00 p.m., CET). After the determination of fasted blood glucose levels by tail vein blood sample collection, animals received an intraperitoneal injection of 20% glucose (10 mL/kg body weight = 2 g glucose/kg body weight) or insulin (0.75 mU insulin/gram body weight; Insuman rapid, Sanofi), respectively. Subsequently, glucose measurements were performed at 0, 15, 30, 60, and 120 min after glucose injection (Figure 1C) and at 0, 15, 30, and 60 min after insulin injection (Supplementary Figure S1), respectively, using an automatic glucose monitor (GlucoMen; A. Menarini Diagnostics, Germany).

### 2.4. Biomarker Analyses

Non-fasted serum levels were analyzed by Milliplex MAP Mouse Adipokine Magnetic Bead Panel—Endocrine Multiplex Assay (#MADKMAG-71K) and Milliplex MAP Mouse Aging Magnetic Bead Panel 1 (#MAGE1MAG-25K) (Merck Millipore) following the manufacture’s guidelines for the following markers: interleukin-6 (IL-6), insulin, leptin, and monocyte chemoattractant protein 1 (MCP-1). Using the median fluorescence intensity and the standard curve, the absolute concentration of each cytokine (pg/mL) was calculated (Bio-Plex Manager 6.1; Bio-Rad Laboratories). Probes are included if they are within the manufacture’s minimum detectable concentration (IL-6: 2.3 ± 6.3 pg/mL, insulin: 13.0 ± 27.7 pg/mL, leptin: 4.2 ± 8.2 pg/mL, and MCP-1: 4.9 ± 11.9 pg/mL).

### 2.5. Quantitative Real-Time Polymerase Chain Reaction (qRT-PCR)

qRT-PCR was performed as previously described [29,33,34,35,36]. Briefly, total ribonucleic acid (RNA) was isolated from liver tissue using TriReagent^®^ (Sigma-Aldrich, Steinheim, Germany) according to the manufacturer’s guidelines. RNA quantity and purity were determined by measuring UV absorption with a NanoDrop spectrophotometer (Nano Quant infinite M200 Pro) and RNA was then converted to cDNA. Quantitative changes in mRNA expression were determined by qRT-PCR as previously described [33,35,36], using the 7500 real-time PCR system (Applied Biosystems, Foster City, CA, USA). Primer pairs and Taqman probes are listed in Supplementary Table S1. The relative expression levels of each gene were calculated in comparison to the corresponding control genes (glyceraldehyde 3-phosphate dehydrogenase (*Gapdh*) and glucuronidase-ß (*Gusb*)) that were used for normalization of expression.

### 2.6. Protein Isolation

For protein isolation, frozen liver samples were homogenized and mixed with protein extraction buffer (6.65 mol/L Urea, 10% Glycerol, 1% Sodium dodecyl sulfate [SDS], 10 mmol/L Tris-HCl pH 6.8, 5 mmol/L Dithiothreitol, and 0.5 mmol/L Phenylmethanesulfonylfluoride) as previously described [29,33,34,36]. Concentration was determined using a Bicichonin acid (BCA)-Protein Assay Kit (Thermo Scientific, Waltham, MA, USA).

### 2.7. Immunoblotting

Immunoblotting of liver samples was performed as previously described [33,34,36,37]. For quantitative immunoblot analysis, densitometry was performed using Bio-Rad ImageLab software (Bio-Rad, Munich, Germany). Protein samples were normalized by using GAPDH as the loading control from the same samples. Original blots are provided in the Supplementary Material. Primary antibodies are listed in Supplementary Table S2. For detailed information, see Supplementary Methods.

### 2.8. Histological Analysis of the Liver Tissue

Histological analysis of liver samples was performed as previously described [29]. Briefly, upon sacrifice, the liver tissue was excised and immediately fixed in 4% paraformaldehyde. Liver samples were embedded in paraffin and sectioned at 3 µm. Slices were stained with hematoxylin and eosin (H&E) for microscopic examination and the degree of steatosis, the type of steatosis, lobular inflammation and hepatocellular ballooning were evaluated. To quantitatively assess the hepatic effect caused by exercise and dietary intervention, grade of steatosis, inflammation, ballooning, and fibrosis were scored and NAFLD-activity score (NAS) was calculated as previously described [38,39,40]. The sections and the light microscopy were evaluated by an expert liver pathologist, who was blinded to the exercise and dietary conditions.

### 2.9. Statistical Analysis

All values are expressed as mean ± standard deviation (SD). The real-time RT-PCR results were calculated based on the ΔΔ-Ct method and expressed as fold induction of mRNA expression compared with the corresponding control group (1.0-fold induction) as previously described [29,33]. For the comparison of measurements between the two groups, we performed an unpaired t-test for parametric distribution or Mann–Whitney t-tests for nonparametric distribution. For a quantitative comparison between time points, a two-way ANOVA followed by the Bonferroni post-test was performed. Statistical significance was defined as *p* < 0.05. The statistical analysis was performed using the Graph Pad Prism software (GraphPad version 8.0, San Diego, CA, USA).

## 3. Results

### 3.1. ME before and during Pregnancy Induces an Altered Body Composition in Obese Dams with a Reduced Amount of Epigonadal Fat Mass

To investigate the direct effects of maternal exercise at an obese state, we first established a diet-induced obesity cohort of dams. Following a seven-week WSD feeding period, the maternal obesity cohort had increased body weight (Figure 1B, *p* < 0.001) and revealed impaired glucose tolerance compared to standard diet-fed controls at week 10 (Figure 1C, *p* < 0.05), indicating an impaired metabolic phenotype. After subsequent randomization to either the voluntary wheel running group (WSD-Run) or sedentary life-style group (WSD), there was no significant difference in maternal weight gain during pregnancy (Figure 1D). 

In the WSD-Run group, the average running distance initially increased from about 13 to 16 km/day before pregnancy. At the beginning of pregnancy, the average running distance was about 6.7 km/day and then progressively decreased when the obese–pregnant mice approached delivery to less than 900 m/day (Figure 1E). Following the exercise intervention, there was no significant difference in litter size (Supplementary Figure S1). At the end of lactation (P21), dams of both groups showed no differences in terms of total body weight (Figure 1F), while exercised obese dams revealed a significantly reduced mass of epigonadal adipose tissue when compared to sedentary obese dams (Figure 1G, *p* < 0.01).

### 3.2. ME Prevents Obese Dams from WSD-Induced Hepatic Steatosis 

**Figure 2:**
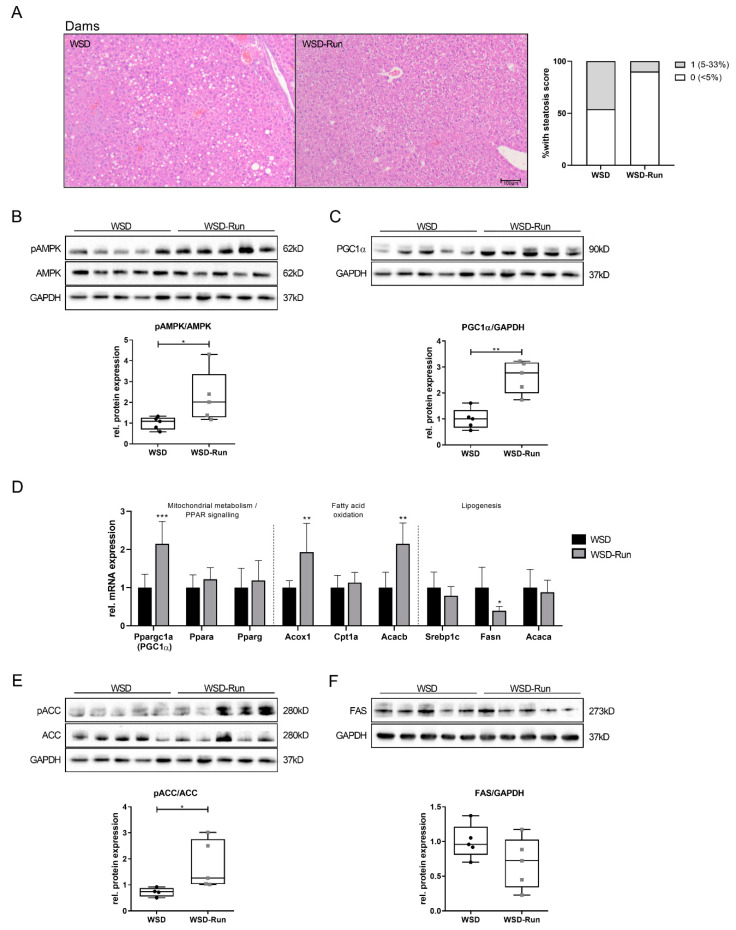
Maternal exercise before and during pregnancy prevents obese dams from WSD-induced hepatic steatosis. (**A**) Histological hepatic steatosis in dams at the end of lactation (P21) by H&E staining. Magnification: 20×. Percentage of steatosis, scored as 0 (<5%) or 1 (5–33%) percentage of hepatocytes containing lipid droplets (WSD group (n = 13), WSD-Run group (n = 9)). (**B**,**C**) Assessment of key hepatic energy regulators using immunoblots. (**B**) pAMPK/AMPK protein expression. (**C**) PGC1α protein expression. (**D**) Assessment of regulators of hepatic mitochondrial metabolism (*Ppargc1a* (Pgc1α), *Ppara*, *Pparg*), hepatic fatty acid oxidation (*Acox1*, *Cpt1a*, *Acacb*), and hepatic lipogenesis (*Srebp1c*, *Fasn*, *Acaca*) by qPCR (WSD group (n = 5), WSD-Run (n = 10)). (**E**,**F**) Assessment of key enzymes of hepatic lipogenesis using immunoblots. (**E**) pACC/ACC protein expression. (**F**) FAS protein expression. Representative immunoblots are presented above the respective graph. Immunoblots: WSD group (n = 5), WSD-group (n = 5). GAPDH served as loading control. Data are presented as mean ± standard deviation; * *p* < 0.05, ** *p* < 0.01, *** *p* < 0.001. ACC, acetyl-CoA carboxylase; Acac, gene encoding for acetyl-CoA carboxylase; Acox, acyl-CoA-oxidase; Cpt1a, carnitine palmitoyltransferase 1a; FAS, fatty acid synthase; Fasn, gene encoding for fatty acid synthase; GAPDH, glyceraldehyde 3-phosphate dehydrogenase; Ppara/g, peroxisome proliferator-activated receptors α/γ; p, phosphorylated; qPCR, quantitative real-time polymerase chain reaction; Srebp1c, sterol regulatory element-binding protein-1c.

To evaluate the effects of ME before and during pregnancy on hepatic lipid content, the liver histomorphology of dams was analyzed following lactation at P21. Exercised obese mothers were mainly protected against WSD-induced hepatic steatosis and showed reduced hepatic fat deposition compared to the sedentary lifestyle group (Figure 2A). 

Next, we determined the effects of ME on AMPK as an essential regulator of hepatic energy metabolism. Dams of the WSD-Run group revealed a significant increase in hepatic phosphorylated (p) AMPK compared to sedentary WSD dams (Figure 2B, *p* < 0.05). PGC1α (Pppargc1-α) represents a major downstream effector of AMPK and is known to be a master regulator of hepatic mitochondrial biogenesis [23]. PGC1α expression was significantly increased on mRNA and protein levels in WSD-Run dams compared to the sedentary WSD group, indicating an increased activity of the AMPK-PGC1α signaling pathway (Figure 2C, *p* < 0.01; 2D, *p* < 0.001). As AMPK and PGC1α are known to regulate hepatic β-oxidation, Acox1 (acyl-coenzyme A oxidase 1), Cpt1a (carnitine palmitoyltransferase 1A) and Acacb (acetyl-CoA carboxylase 2) as marker of β-oxidation, were assessed [41]. Acox1 and Acacb mRNA levels were significantly increased in WSD-Run dams (Figure 2D, *p* < 0.01), while mRNA levels of Cpt1a were not significantly altered by ME. 

Next, we focused on AMPK downstream targets of the hepatic lipogenesis, namely ACC (acetyl-CoA-carboxylase) and FAS (fatty acid synthase) by Western blot. AMPK regulates hepatic lipid metabolism through downregulation of FAS and phosphorylation of ACC, leading to an inhibition of hepatic de novo lipogenesis and stimulation of fatty acid oxidation, respectively. Both processes counteract the development of hepatic steatosis and are dysregulated in the pathogenesis of metabolic dysfunction associated fatty liver disease. Here, we observed a significant increase in phosphorylated ACC protein levels in exercised obese dams compared to sedentary controls (Figure 2E, *p* < 0.05). While Fasn mRNA levels were significantly downregulated in WSD-Run dams compared to WSD dams (Figure 2D, *p* < 0.05), protein levels were not significantly altered (Figure 2F). Srebp1c (sterol regulatory element-binding protein 1c), Acaca (acyl-coenzyme A oxidase 1) and Ppara (peroxisome proliferator-activated receptor α) differed not between the groups (Figure 2D). 

**Figure 3:**
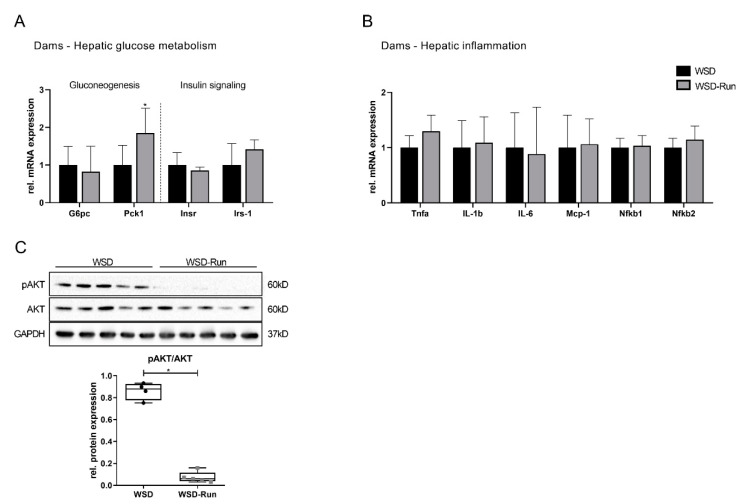
Impact of maternal exercise on markers of hepatic glucose metabolism and inflammation. (**A**) Assessment of regulators of hepatic gluconeogenesis (*G6pc*, *Pck1*) and hepatocellular insulin signalling (*Insr*, *Irs-1*) by qPCR (WSD group (n = 5), WSD-Run (n = 10)). (**B**) Determination of regulators of hepatic inflammation (*Tnfa*, *Il-1b*, *Il-6*, *Mcp-1*, *Nfkb1*, *Nfkb2*) by qPCR (WSD group (n = 5), WSD-Run (n = 10)). (**C**) Representative immunoblot of phosphorylated (p)AKT, as marker of hepatic insulin signalling (WSD group (n = 5), WSD-Run group (n = 5)). GAPDH served as loading control. Data are presented as mean ± standard deviation; * *p* < 0.05. AKT, protein kinase B; GAPDH, glyceraldehyde 3-phosphate dehydrogenase; G6pc, glucose-6-phosphatase; Insr, insulin receptor; Irs-1, insulin receptor substrate-1; Il-1b, interleukin-1b; Il-6, interleukin-6; Mcp-1, monocyte chemoattractant protein-1; Nfkb, nuclear factor kappaB; Pck1, phosphoenolpyruvate carboxykinase; Tnfa, tumor necrosis factor-alpha.

The additional investigation of hepatic glucose metabolism revealed an elevated expression of essential gluconeogenic genes, such as those encoding for phosphoenolpyruvate carboxykinase (Pck1) and glucose-6-phosphatase (G6pase) (Figure 3A, *p* < 0.05), while the phosphorylation of AKT, as a marker of hepatic insulin action, was significantly downregulated in exercised obese dams (Figure 3C, *p* < 0.05). Markers of hepatic inflammation showed no difference in expression between the sedentary and exercised dams (Figure 3B).

### 3.3. ME Exerts No Influence on Offspring’s Body Weight and Body Composition at P21

**Figure 4:**
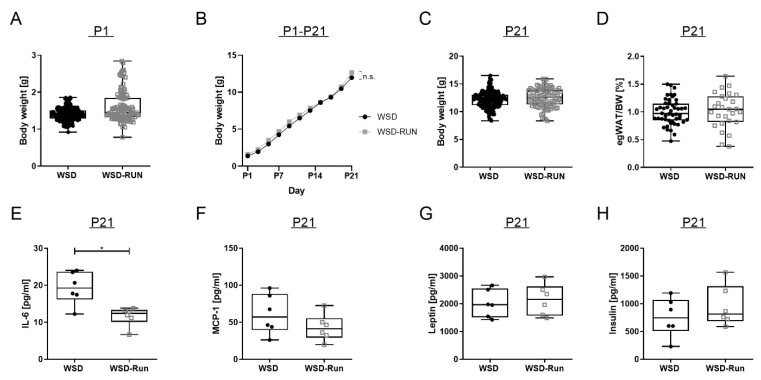
Maternal exercise before and during pregnancy shows no influence on offspring’s body weight and body composition in early life (P21). (**A**) Offspring total body weight a birth (P1). (**B**) Offspring body weight gain during the lactation period (P1–P21). (**C**) Offspring total body weight at weaning at postnatal day P21 (WSD group (n = 179), WSD-Run group (n = 94)). (**D**) Offspring epigonadal fat pad weight at P21 (WSD group (n = 49), WSD-group (n = 28)). (**E**–**G**). Serum analyses of IL-6 (**E**), MCP-1 (**F**), leptin (**F**) and insulin, (**F**) using multiplex assays in offspring at weaning (P21) (WSD group (n = 6), WSD-group (n = 6)). Data are presented as mean ± standard deviation; * *p* < 0.05. IL-6, interleukin 6; MCP-1, monocyte chemoattractant protein-1; *p*, postnatal day.

**Figure 5:**
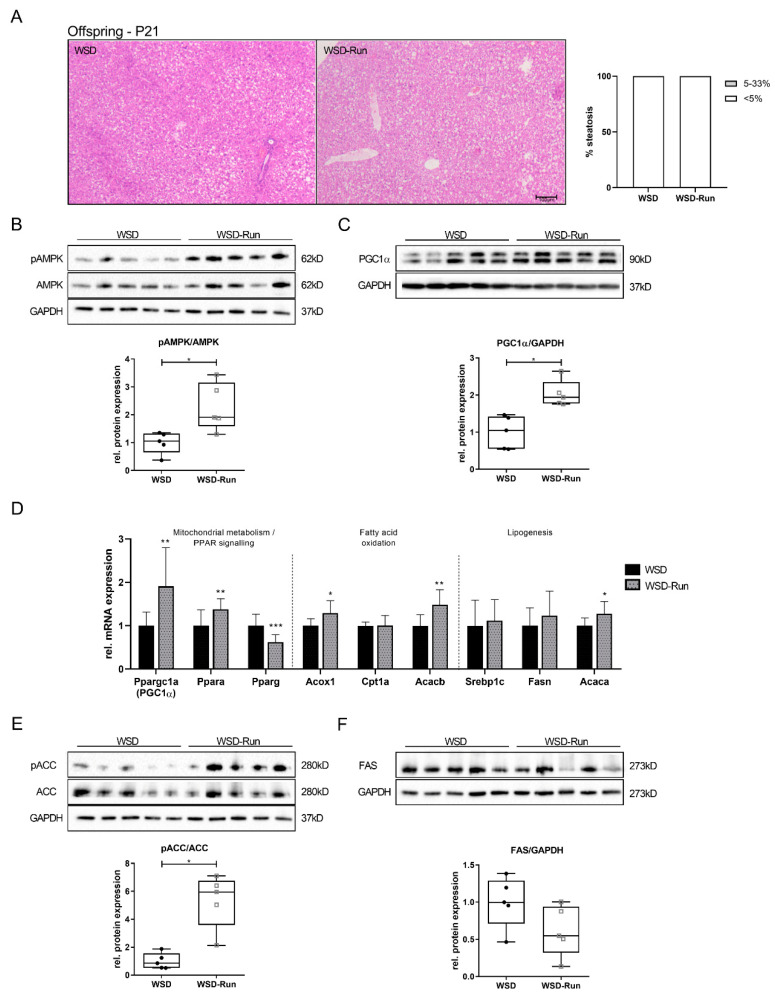
Maternal exercise before and during pregnancy alters offspring hepatic AMPK-PGC1α axis in early life (P21). (**A**) Histological analysis of amount of hepatic steatosis in offspring at the end of lactation (P21) by H&E staining revealed no significant hepatic steatosis in offspring of both groups in early life. Magnification: 20×. Percentage of steatosis, scored as 0 (< 5%) percentage of hepatocytes containing lipid droplets (WSD group (n = 19), WSD-Run group (n = 12)). (**B**,**C**) Assessment of key hepatic energy regulators using immunoblots revealed an expression profile comparable to that observed in the dams. (**B**) pAMPK/AMPK protein expression. (**C**) PGC1α protein expression. (**D**) Assessment of regulators of hepatic mitochondrial metabolism (*Ppargc1a* (Pgc1α), *Ppara*, *Pparg*), hepatic fatty acid oxidation (*Acox1*, *Cpt1a*, *Acacb*), and hepatic lipogenesis (*Srebp1c*, *Fasn*, *Acaca*) by qPCR (WSD group (n = 10), WSD-Run (n = 10)). (**E**,**F**) Determination of markers of hepatic lipogenesis using immunoblots. (**E**) pACC/ACC protein expression. (**F**) FAS protein expression. Representative immunoblots are presented above the respective graph. Immunoblots: WSD group (n = 5), WSD-group (n = 5). GAPDH served as loading control. Data are presented as mean ± standard deviation; * *p* < 0.05, ** *p* < 0.01, *** *p* < 0.001. ACC, acetyl-CoA carboxylase; Acac, gene encoding for acetyl-CoA carboxylase; Acox, acyl-CoA-oxidase; Cpt1a, carnitine palmitoyltransferase 1a; FAS, fatty acid synthase; Fasn, gene encoding for fatty acid synthase; GAPDH, glyceraldehyde 3-phosphate dehydrogenase; Ppara/g, peroxisome proliferator-activated receptors α/γ; p; phosphorylated; qPCR, quantitative real-time polymerase chain reaction; Srebp1c, sterol regulatory element-binding protein-1c.

To determine the effects of maternal exercise on offspring body weight and body composition in their early life, offspring body weight was measured every second day during the lactation period (P1–P21). Comparing WSD and WSD-Run offspring at weaning (P21), there was no significant difference in the offspring body weight or epigonadal fat pad weight (Figure 4C and D). Serum leptin, insulin and MCP-1 levels revealed no significant difference at P21 (Figure 4F–H). Interleukin-6 (IL-6) is a vital adipocytokine in mediating low-grade inflammation effects in obese individuals. Circulating IL-6 serum levels were significantly decreased in the offspring of exercised dams compared to the offspring of sedentary dams (Figure 4E, *p* < 0.05). There were no signs of relevant hepatic steatosis in either group at P21 (Figure 5A).

### 3.4. Offspring of Exercised Obese Dams Exhibit an Increased Hepatic AMPK-PGC1α Axis in Early Life

To analyze the specific effects of ME on the hepatic metabolism in the offspring, we next focused on critical regulators of hepatic energy metabolism, namely AMPK and PGC1α (Figure 5B,C). In line with the dams, significantly increased pAMPK protein expression and significantly increased PGC1α mRNA and protein expression levels could be detected in the offspring of exercised obese dams at P21, even though the offspring did not perform an exercise intervention themselves (Figure 5B and C, *p* < 0.05). This increase in AMPK-PGC1α axis activity in early life was accompanied by increased mRNA expression of Ppra (PPARα) in WSD-Run offspring (Figure 5D, *p* < 0.01). Activation of PPARα promotes the uptake, utilization, and catabolism of fatty acids by upregulation of genes involved in fatty acid transport (Cpt1a), fatty acid binding (Acacb), and hepatic peroxisomal and mitochondrial fatty acid β-oxidation (Acox1) [42,43]. ME significantly increased Acox1 and Acacb mRNA levels in WSD-Run offspring, while Cpt1a levels were not altered (Figure 5D, Acox1: *p* < 0.05, Acacb: *p* < 0.01). Elevated peroxisome proliferator-activated receptor gamma (PPARy) levels in the liver are associated with hepatic steatosis, while hepatocyte specific disruption of PPARy (Pparg) gene expression decreased liver steatosis in ob/ob mice [43,44,45]. Thus, we next determined Pparg mRNA expression in offspring and observed a significant decrease in WSD-Run offspring compared to sedentary controls (Figure 5D, *p* < 0.001).

Focusing on major downstream targets of AMPK regulating hepatic lipogenesis, a significantly increased expression of pACC (Figure 5E, *p* < 0.05), indicating inhibition of hepatic lipogenesis, could be observed in the offspring of exercised dams, while FAS levels showed a tendency to be downregulated (Figure 5F). Hepatic mRNA levels of Srebp1c were not altered in the offspring by ME (Figure 5D).

**Figure 6:**
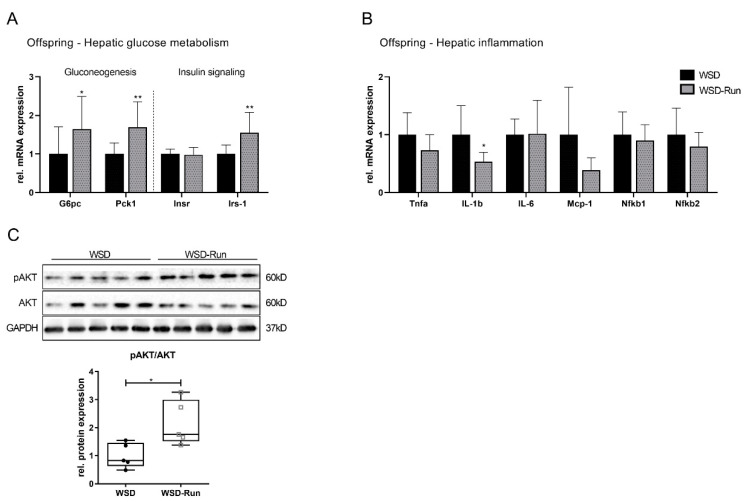
Impact of maternal exercise on offspring hepatic glucose metabolism and hepatic inflammation in early life (P21). (**A**) Assessment of regulators of hepatic gluconeogenesis (*G6pc*, *Pck1*) and hepatocellular insulin signaling (*Insr*, *Irs-1*) by qPCR (WSD group (n = 10), WSD-Run (n = 10)). (**B**) Determination of regulators of hepatic inflammation (*Tnfa*, *Il-1b*, *Il-6, Mcp-1*, *Nfkb1*, *Nfkb2*) by qPCR (WSD group (n = 10), WSD-Run (n = 10)). (**C**) Representative immunoblot of (p)AKT, as marker of hepatic insulin signaling (WSD group (n = 5), WSD-group (n = 5)). GAPDH served as the loading control. Data are presented as mean ± standard deviation; * *p* < 0.05, ** *p* < 0.01. AKT, protein kinase B; GAPDH, glyceraldehyde 3-phosphate dehydrogenase; G6pc, glucose-6-phosphatase; Il-1b, Insr, insulin receptor; Irs-1, insulin receptor substrate-1; Interleukin-1b; Il-6, interleukin-6; Mcp-1, monocyte chemoattractant protein-1; Nfkb, nuclear factor kappaB; Pck1, phosphoenolpyruvate carboxykinase; Tnfa, tumor necrosis factor-alpha.

Analysis of hepatic glucose metabolism in the offspring revealed increased expression of markers of hepatic gluconeogenesis (G6pc, Pck1) (Figure 6A, G6pc: *p* < 0.05; Pck1: *p* < 0.05). At the same time, an increased mRNA expression of insulin receptor substrate (Irs) 1 (Figure 6A) and an increased protein expression of pAKT were found in offspring of exercised obese dams (Figure 6C, *p* < 0.05). Taken together, our findings suggest a favorable constellation of hepatic lipid metabolism in offspring of exercised obese dams in early life marked by activation of the AMPK- PGC1α axis at P21.

### 3.5. Offspring of Exercised Obese Dams Were Protected from WSD Induced Hepatic Steatosis and Increase in Adipose Tissue Mass in Later Life

**Figure 7:**
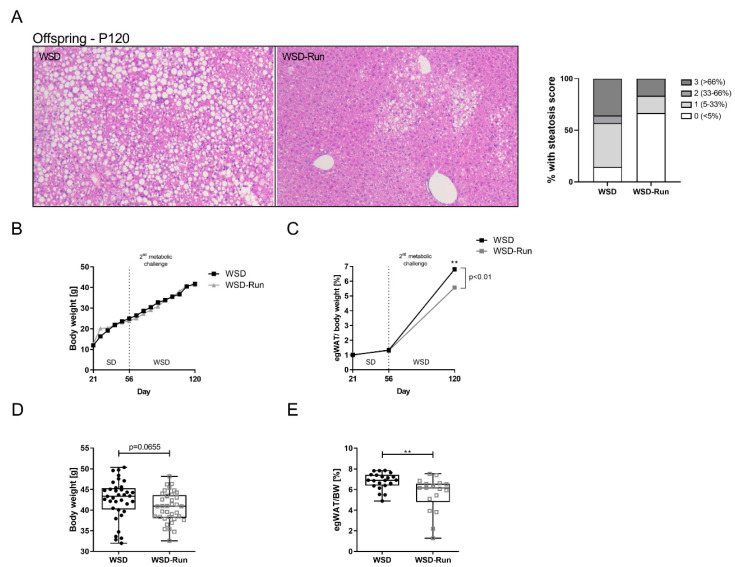
Offspring of exercised obese dams were protected from WSD induced hepatic steatosis and increase in adipose tissue mass in later life (P120). (**A**) Histological analysis of amount of hepatic steatosis after offspring were re-exposed to WSD over a 9-week period later in life. H&E staining, magnification: 20×. Percentage of steatosis, scored as 0 (<5%); 1 (5–33%); 2 (34–66%) and 3 (>66%) percentage of hepatocytes containing lipid droplets (WSD group (n = 14), WSD-Run group (n = 12)). (**B**) Total body weight gain of offspring while re-challenged with WSD from P56 until P120. (**C**) Increase in epigonadal fat mass of the offspring after re-exposure to WSD. (**D**) Total body weight after 9 weeks of WSD at P120 (WSD group (n = 24), WSD-Run group (n = 22)). (**E**) Epigonadal fat pad weight at P21 (WSD group (n = 22), WSD-Run group (n = 18)). Data are presented as mean ± standard deviation; ** *p* < 0.01.

To examine long-term preventive effects of ME, offspring were re-exposed to an obesogenic diet for nine weeks starting at P56 to mimic a metabolic challenge later in life. Within this period, weight progression was measured weekly, followed by an analysis of body weight, epigonadal fat mass and liver phenotype at P120. Upon the re-start of WSD-feeding at P56, both groups showed a substantial, comparable weight gain of over 65% compared to baseline at P56 (WSD: +70.44%; WSD-Run: +66.12%) (Figure 7B). While total body weight only tended to be lower in the offspring of exercised obese dams at P120 (Figure 7B; mean body weight, 40.70 g (WSD) versus 42.56 g (WSD-Run), *p* = 0.0655), WSD-Run offspring were protected against the significant WSD-induced increase in adipose tissue mass as seen in WSD offspring (Figure 7C and E, *p* < 0.01). Accordingly, WSD-Run offspring showed markedly reduced hepatic steatosis, when compared to offspring of sedentary obese dams (Figure 7A). 

Thus, offspring of exercised obese dams were protected from WSD-induced hepatic steatosis and increased adipose tissue mass in later life.

## 4. Discussion

Accumulating evidence indicates that an adverse early life environment can have a long-term impact on offspring metabolic health. While adverse causative factors, such as maternal overnutrition or sedentary lifestyle, have been clearly identified, it is also of particular relevance to improve the understanding of preventive measures and their underlying molecular mechanisms to identify new potential therapeutic approaches.

To this end, we implemented an exercise intervention of voluntary wheel running into a critical period of developmental programming and investigated to what extent adverse metabolic effects of maternal obesity could be counteracted. We focused in this study on the liver as a central regulator of metabolic homeostasis and on hepatic AMPK signalling as a potential therapeutic target. In addition to the direct effects on maternal hepatic metabolism, the short- and long-term effects of maternal exercise on the offspring’s hepatic metabolism were also investigated. 

In the dams, exercised obese dams presented less hepatic steatosis when compared to the sedentary group. This was accompanied by an increase in pAMPK and PGC1α signaling. Under conditions of energetic stress, e.g., due to fasting or physical exercise, AMPK is an essential metabolic sensor and has regulating effects on hepatic fatty acid synthesis, lipolysis, ß-oxidation, and glucose homeostasis [18,23,41]. AMPK is also critically involved in regulating mitochondrial homeostasis, in particular, via PGC1α, an important downstream effector and key mediator of mitochondrial biogenesis [46]. PGC1α is a powerful regulator of various metabolic pathways and has substantial involvement in several diseases characterized by energetic misbalance, such as NAFLD. While PGC1α expression is blunted in the steatotic liver, high levels of hepatic PGC1α might ameliorate NAFLD [46,47]. Therefore, we determined PGC1α and could see a substantial increase in WSD-Run dams, indicating an exercise-induced activation of the AMPK-PGC1α axis.

Upon exercise, particularly, hepatic fatty acid β-oxidation is activated for ATP generation, while energy-consuming lipogenesis is inhibited simultaneously [46,48]. Both AMPK and PGC1α can regulate the expression of genes involved in hepatic fatty acid oxidation and hepatic lipogenesis, respectively.

AMPK phosphorylation of acetyl-CoA carboxylases (ACC1 and ACC2) has been proposed as a master contributor to changes in lipid metabolism. Upon activation of AMPK, phosphorylation of ACC1 and ACC2 results in the inhibition of ACC activity. This is thought to inhibit the first step in fatty acid synthesis and stimulate lipid β-oxidation in mitochondria [41]. In line with the literature, phosphorylation of ACC and *Acc2* mRNA expression were significantly increased in WSD-Run dams, indicating reduced hepatic fatty acid synthesis and increased hepatic β-oxidation, accompanied by less hepatic steatosis [20,49]. Additionally, Acox1, the first enzyme of the fatty acid β-oxidation pathway [50] was significantly increased in exercised dams, indicating increased β-oxidation.

In contrast, CPT1a, the rate-limiting enzyme responsible for shuttling fatty acids into the mitochondrion for fatty acid oxidation, was unaltered. The regulation of CPT1a is complex and has several layers that involve genetic, epigenetic, physiological, and nutritional modulators [51]. Foretz and colleagues recently generated a mouse model of AMPK activation specifically in the liver and observed reduced hepatic lipid content as well as significantly increased fatty acid oxidation that was also independent of changes in *Cpt1a* mRNA expression levels [20]. Taken together, the increase in the AMPK-PGC1α axis observed in exercised obese dams appears to be substantially involved in mediating the reduction in hepatic steatosis via increased mitochondrial fatty acid oxidation and inhibition of lipogenesis.

While ME exhibited substantial effects on the hepatic lipid metabolism of obese dams, there were only minor effects on markers of hepatic inflammation and hepatic glucose metabolism. Regarding gluconeogenesis one marker, Pepck was significantly increased, while G6pc was not altered. Recently, it has been shown that hepatic gluconeogenesis is not directly controlled by AMPK. While early studies using nonspecific AMPK activators (e.g., AICAR and metformin) suggested that AMPK repress gluconeogenesis and glucose production [52,53], recent studies have refuted these initial findings. Using a combination of genetic mouse models lacking AMPK and investigation of direct pharmacological activators of AMPK, it can be shown that AMPK does not inhibit hepatic gluconeogenesis [54,55,56,57]. The increased expression of markers of gluconeogenesis observed in the present study is more likely due to increased mitochondrial metabolism, as previous studies demonstrate that induction of lipid oxidation is required for the endergonic steps of gluconeogenesis. In the case of an increased systemic energy demand, i.e., during exercise, both processes, fatty acid oxidation and gluconeogenesis, are constitutively activated [58,59].

Accumulating evidence indicates that exposure to an adverse in utero environment increases susceptibility to NAFLD in childhood and later life [60,61]. Interestingly, programmed changes in the offspring hepatic metabolism are often already apparent in early stages of life, even if no phenotypic changes can yet be detected [32,62,63,64,65]. Having identified the end of the lactation period (P21, weaning) as a critical window for developmental programming previously [29,35] in which effects of maternal exercise are already detectable on a molecular level, this timepoint was first examined. Molecular analyses at weaning revealed a significant activation of hepatic AMPK and PGC1α signaling in WSD-Run offspring at P21. This was accompanied by increased expression of downstream factors, such as Acc2 and Acox1, indicating increased fatty acid oxidation.

Furthermore, a decrease in hepatic lipogenesis mediated by increased phosphorylation of ACC and a decrease in FAS was found. These adaptations of hepatic metabolism can be interpreted as a protective hepatic signature in the offspring of exercised dams against excessive hepatic fat accumulation. The AMPK-mediated inactivation of ACC was associated with signs of an improved hepatic insulin sensitivity in WSD-Run offspring, illustrated by increased phosphorylation of AKT and significantly elevated mRNA expression levels of *Irs-1*. This interaction has already been shown in previous studies and is consistent with the observation that offspring of exercised dams do not exhibit hepatic steatosis in early life, which is often associated with hepatocellular insulin resistance [66].

As part of their role as central regulators of hepatic energy metabolism and mitochondrial homeostasis, AMPK and PGC1α are critically involved in activating members of the PPAR family [41,46]. *Pparα* mRNA levels were significantly increased in offspring of exercised dams, representing an essential activator of hepatic β-oxidation [43,45]. At the same time, WSD-Run offspring revealed a significant reduction in hepatic PPARγ. Under conditions of nutrient overload and obesity, PPARγ is induced and activated in the liver, where it is involved in fatty acid storage as lipid droplets [43,45]. Overexpression of PPARγ in hepatocytes increased hepatosteatosis, while hepatocyte-specific disruption of *Pparγ* gene expression decreased liver steatosis in ob/ob mice [67]. Taken together, WSD-Run offspring exhibited a similar activity profile of the hepatic AMPK-PGC1α axis as the exercised dams, thus providing a specific signature of hepatic lipid and glucose metabolism that might convey protection from adverse metabolic dysfunction in early life.

The question arises as to what the reason for persistent activation of the AMPK signaling pathway in the offspring at weaning could be. While direct exercise-mediated effects appear to be responsible for AMPK–PGC1α activation in dams, this protective activation appears to be mediated via indirect effects in offspring, as offspring did not undergo any exercise intervention themselves. There are several potential causes for this observation that can be discussed in this context. Offspring of obese mothers display metabolic disorders similar to that observed for maternal undernutrition in the absence of further nutritional insults [61]. This can be attributed to the observation that excessive maternal caloric intake per se may represent a form of fetal malnutrition due to placental dysfunction with altered nutrient transport [61]. Maternal exercise during pregnancy can counteract this and is able to ameliorate placental function and nutrient transport to the fetus, thereby activating AMPK associated signaling pathways [68].

Epigenetic changes could also be responsible for the observed changes in the present study. Epigenetic developmental programming may manifest through changes in DNA methylation, histone modifications, and microRNA expression, whereby hypermethylation is associated with suppressed gene transcription. In accordance, the offspring of obese sedentary dams exhibited PGC1α hypermethylation, decreased PGC1α protein expression levels and increased hepatic steatosis [69,70]. Maternal exercise in obese dams prevented the offspring from PGC1α hypermethylation in the muscle of exercised dams compared to offspring of sedentary dams with concurrent amelioration of systemic glucose metabolism, indicating toward a strong epigenetic effect of exercise on offspring RNA transcriptional regulation [71]. As epigenetics is described as the interaction between the genome and environmental stimuli, it seems possible that physical activity represents such an inducible stimulus, mediating alterations in an individual’s epigenetic profile [72]. In line with this, the altered gene expression of key regulators of lipid metabolism observed in the underlying study could, thus, have been induced as a result of exercise-mediated epigenetic mechanisms.

However, further studies are needed to elucidate the effect of ME on offspring epigenetic modulation of the hepatic AMPK-PGC1α axis. 

In addition to epigenetic changes, alteration in the gut microbiota represents another potential method for passing phenotypes across generations, as an altered microbiome may undergo vertical transmission from dams to offspring [69]. A shift in the offspring’s microbiota due to maternal obesity or maternal obesogenic diet has been reported in both humans and preclinical models [73,74,75,76]. Since maternal exercise in obese dams may improve HFD-induced abnormalities in gut microbiota in offspring [77], and changes in the gut microbiota can in turn be associated with AMPK activation [78,79]; this represents another potential activation mechanism.

Focusing on the offspring in later life, we observed a protection from WSD-induced hepatic steatosis in offspring of exercised obese dams at P120. In line with Stanford et al., total body weight was not altered in the offspring by maternal exercise at that age, but offspring of exercised dams displayed significantly reduced epigonadal fat pad weight, indicating an altered body composition. 

However, as significant changes of hepatic metabolism could already be seen at P21, this clearly indicates that preconditioning for a disturbed hepatic metabolic function takes place already in earlier stages of life. 

Over the past two decades, a number of studies have sought to identify the temporal developmental origins of NAFLD in the context of a maternal obesogenic environment. In rodent studies, offspring born to dams chronically consuming a high-fat diet (HFD) from preconception to lactation displayed severe hepatic steatosis and glucose intolerance in adulthood, despite being fed a standard chow diet after weaning [80,81]. This is in line with findings from nonhuman primates (NHP), where chronic consumption of a HFD prior to and during pregnancy led to fetal liver steatosis, which persisted into the juvenile period [82]. Interestingly, changing the maternal diet in a model of maternal obesity to a low-fat diet several weeks before the onset of pregnancy improved the offspring’s metabolic and hepatic outcome and normalized hepatic free fatty acid composition in later life [83].

Overall, the reported findings emphasize that the periods immediately before the onset of pregnancy, during pregnancy, and lactation are critical windows for programming susceptibility to hepatic metabolic dysfunction. Precisely for this reason, preventive measures should be initiated during these critical periods of developmental programming before detrimental effects are irreversible in later life.

Due to its multiple beneficial effects on both hepatic and whole-body metabolism, AMPK represents a promising drug target for treating chronic metabolic diseases [23,41]. Drugs that can induce either direct or indirect activation of AMPK include metformin, salicate and canagliflozin, a sodium–glucose cotransporter 2 (SGLT2) inhibitor that has been recently approved for the treatment of type 2 diabetes [23,41,84]. Furthermore, in recent years, potent and specific small-molecules have been identified and tested in preclinical models, which act as direct allosteric activators of AMPK, and were able to improve glucose homeostasis, reduce lipid levels and inhibit lipid accumulation in the liver [23,41,54,85,86,87]. 

Metformin inhibits complex I in the mitochondria, leading to a reduction in mitochondrial respiration and ATP production, and has been shown to activate AMPK effectively [53]. In addition to its use in treating type 2 diabetes mellitus, metformin is currently also being increasingly used for treating gestational diabetes, as randomized controlled trial evidence is emerging, demonstrating its safety and efficacy during pregnancy [88,89,90]. Since metformin is able to prevent diabetes in pre-diabetic populations and may reduce gestational weight gain in women with moderate-to-severe obesity with and without diabetes, it is currently also being intensively discussed as a preventive therapy option for overweight pregnant women in order to improve metabolic dysfunction and prevent associated negative consequences for the offspring. 

Targeting PGC1α represents another appealing strategy, as it also orchestrates essential aspects of liver homeostasis, such as mitochondrial oxidative phosphorylation, gluconeogenesis and fatty acid synthesis [46]. However, targeting PGC1α is very difficult. Coactivators are hard to target because they lack highly specific ligand binding domains, have large and flexible structures and are localized to the nucleus [46,91]. However, pharmacological modulation of transcriptional and post transcriptional activators of PGC1s to increase their expression might be a viable therapeutic approach to treat metabolic dysfunction. 

The study and the animal model on which it is based have some limitations that should be taken into account when interpreting the study results. First, phenotyping and molecular analyses were only performed in male offspring. Since gender-specific differences in hepatic metabolism as a result of metabolic programming appear possible (e.g., mediated by hormones), future studies should examine and compare both male and female offspring [31]. The missing measurement of direct hepatocellular β-oxidation capacity, circulating free fatty acids and energy consumption assays (e.g., indirect calorimetry) remain further limitations and should be conducted in future studies. 

Even though translation from rodent studies to humans should be discussed critically, these results underline the importance of health programs for obese pregnant women, especially in those who suffer from further metabolic disorders, such as diabetes, and emphasize the need for time- and cost-intensive long-term mother–child studies to examine the effects of ME to offspring hepatic metabolism in men.

## 5. Conclusions

The present study demonstrates that maternal exercise provides a promising strategy to improve the metabolic health of both, the obese mother and her offspring by ameliorating hepatic steatosis and stimulating the AMPK–PGC1α axis. These data highlight AMPK as a potential metabolic target for therapeutic intervention in order to combat the global burden of obesity and hepatic steatosis, especially for the generations to come.

## Data Availability

The data presented in this study are available on request from the corresponding author.

## Supplementary Materials

The following are available online at https://www.mdpi.com/article/10.3390/cells10051247/s1, Supplemental Figure S1: (A) Litter size at P1, (B) Intraperitoneal insulin tolerance test of the dams; Supplemental Figure S2: Animal model; Supplemental Table S1: List of real-time qRT-PCR primer sets; Supplemental Table S2: List of antibodies used for immunoblots; Supplemental Table S3: Information on the composition of the study diet.
