# Supplementary material for: Maternal Exercise Mediates Hepatic Metabolic Programming via Activation of AMPK-PGC1α Axis in the Offspring of Obese Mothers
Source: Cells. 2021 May 19;10(5):1247. doi: 10.3390/cells10051247 (PMC8158724; doi:10.3390/cells10051247)
Supplement: Supplementary file 1 [file cells-10-01247-s001.zip › cells-1192951-supplementary.pdf]

Supplementary Material

Maternal Exercise Mediates Hepatic Metabolic Programming via Activation of AMPK-PGC1α Axis in the Offspring of Obese Mothers

Philipp Kasper [1,†], Saida Breuer [2,†], Thorben Hoffmann [2], Christina Vohlen [2], Ruth Janoschek [2], Lisa Schmitz [2], Sarah Appel [2], Gregor Fink [2], Christoph Hünseler [2], Alexander Quaas [3], Münevver Demir [4], Sonja Lang [1,5], Hans-Michael Steffen [1], Anna Martin [1], Christoph Schramm [1], Martin Bürger [1], Esther Mahabir [6], Tobias Goeser [1], Jörg Dötsch [2], Eva Hucklenbruch-Rother [2,‡] and Inga Bae-Gartz [2,‡,*]

1. Supplementary Figure S1

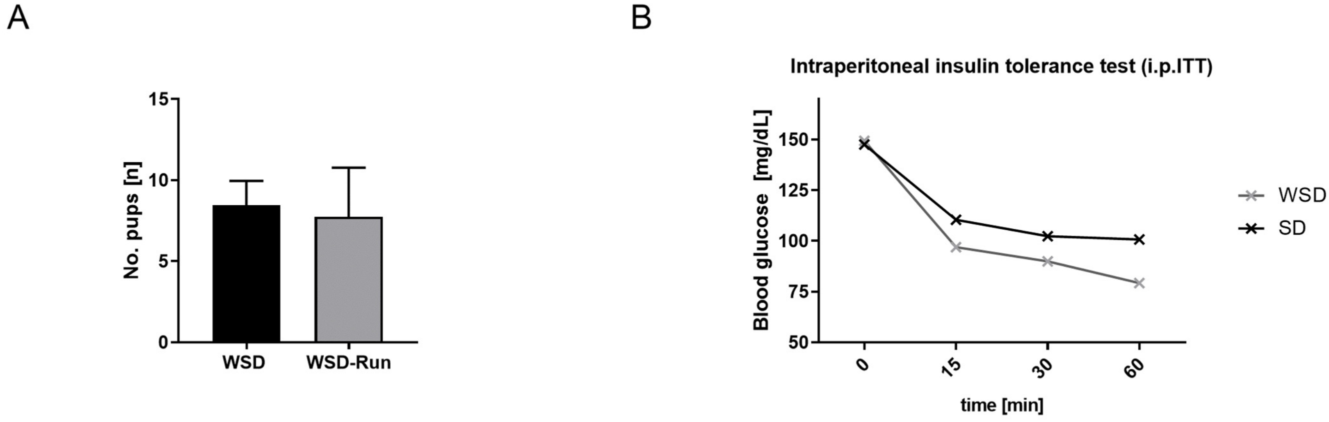

Figure S1. (**A**) Liter size at birth (Mean liter size WSD group: 8.4 pups/dam; mean liter size WSD-

2. Supplementary Figure S2–Animal Model

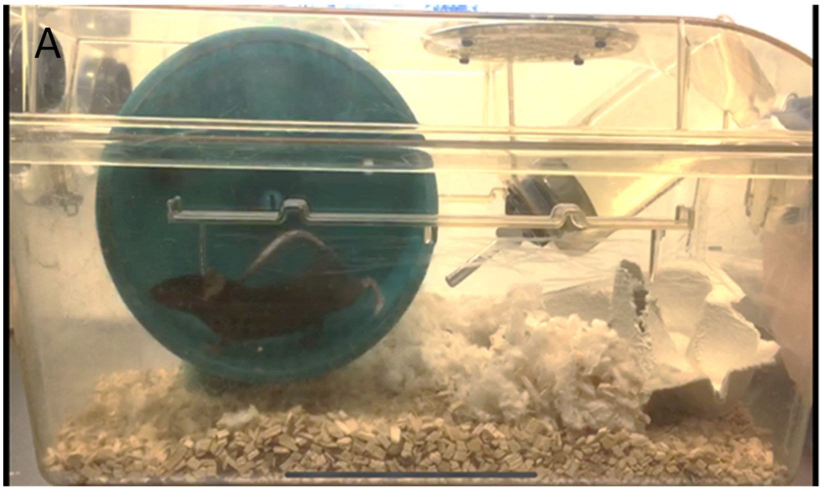

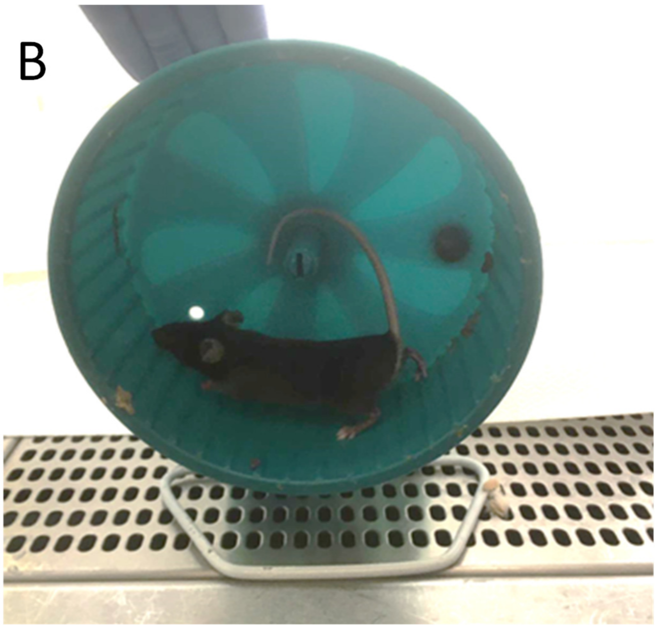

Figure 2. Illustration of the experimental setting and the running wheel provided to the mice for voluntary-wheel-running exercise. Representative image inside the cage (**A**) and for a better overview outside the cage (**B**).

Voluntary wheel running (VWR) was used to examine the mechanisms by which maternal exercise confers improvement on both dams and offspring metabolic health. As illustrated, female C57BL/6N mice were housed individually in cages equipped with a running wheel (WSD-Run group). The running wheel was equipped with a tachometer measuring distance (km), average speed (km/h) and time (h:m). Using this equipment an accurate recording of physical activity for each individual animal was possible. Control animals remained sedentary (WSD group).

3. Supplementary Table S1 (Primer List, Real Time RT-PCR)

Table S1. List of primers used for real-time RT-PCR.

Gene	Primer	Primer sequence 5′-3′
Acaca[1] (Acetyl-CoA carboxylase 1)	for<br>rev	GAAGTCAGAGCCACGGCACA<br>GGCAATCTCAGTTCAAGCCAGTC
Acacb[1] (Acetyl-CoA carboxylase 2)	for<br>rev	CTACAAGACGGCGCAGGTCA<br>AGGCGCCAAACTTCAGCATC
Acox1[1] (Acyl-CoA Oxidase 1)	for<br>rev	GAGCCTTTGGACCTTCACTTGG<br>CGCATAAGTGCCCGTGATCT
Cpt1a[1] (Carnitine Palmitoyltransferase 1A)	for<br>rev	CATTACAAGGACATGGGCAAGTT<br>CCGTAGTGCAGGAGCGTACA
Fasn[1] (Fatty acid synthase)	for<br>rev	CCATGGAGCGTATATGTGAACAG<br>AATGCCCACGTCACCAATG
Gapdh	for<br>rev<br>Probe (FAM, TAMRA)	ATGTGTCCGTCGTGGATCTGA<br>TGCCTGCTTCACCACCTTCT<br>CCGCCTGGAGAAACCTGCCAAGTATG
Gusb	for<br>rev<br>Probe (FAM, TAMRA)	CGCTGAGAGTAATCGGAAACAA<br>CGCAAAATAAAGGCCGAAGT<br>ATCTTCACTCGCCAGAGACAGCCCA
G6pc[1] (Glucose-6-phosphatase)	for<br>rev	TGTCTGTGATTGCTGACCTG<br>GTAGAAGTGACCATAACATAG
Il6 (Interleukin 6)	for<br>rev	ACAAGTCGGAGGCTTAATTACACAT<br>AATCAGAATTGCCATTGCCCAA

	Probe (FAM, TAMRA)	TCTTTTCTCATTTCCACGATTTCCCAGA-GAA
Il1b[1] (Interleukin 1 beta)	for<br>rev	TGACAGTGATGAGAATGACCTGTTC<br>GGACAGCCCAGGTCAAAGG
Insr[1] (Insulin receptor)	for<br>rev	GCAAACAGATGCCACTAATCCTT<br>GGGCTTCCACTTTAAGATAATCTGA
Irs-1[1] (Insulin Receptor Substrate 1)	for<br>rev	CCAGAGTCAAGCCTCACACA<br>CCCAACTCAACTCCACCACT
Mcp-1 (Monocyte chemoattractant protein 1)	for<br>rev<br>Probe (FAM, TAMRA)	GGCTCAGCCAGATGCAGTTAAC<br>CTTGGTGACAAAAACTACAGCTTCTT<br>CCCCACTCACCTGCTGCTACTCATTCA
Nfkb1[1] (Nuclear Factor Kappa B Subunit 1)	for<br>rev	CATCCCGGAGTCACGAAATC<br>GCACAATCTTTAGGGCCATTTT
Nfkb2[1] (Nuclear Factor Kappa B Subunit 2)	for<br>rev	TCTAGCCACAGAGATGGAGGAGTT<br>AGGTCCGGGCATTCACATTA
Pck[1] (Phosphoenolpyruvate carboxykinase, Pepck)	for<br>rev<br>Probe (FAM, TAMRA)	CCACAGCTGCTGCAGAACAC<br>GAAGGGTCGCATGGCAAA<br>AGGGCAAGATCATCATGCACGACCC
Ppara (Peroxisome Proliferator Activated Receptor α)	for<br>rev<br>Probe (FAM, TAMRA)	CCAGTACTGCCGTTTTCACAAGT<br>GCTTTTTCAGATCTTGGCATTCTT<br>TCTGTCGGGATGTCACACAATGCAATTC
Pprag (Peroxisome Proliferator Activated Receptor γ)	for<br>rev<br>Probe (FAM, TAMRA)	CCCAATGGTTGCTGATTACAAA<br>GCCTGTTGTAGAGCTGGGTCTT<br>ACCTGAAGCTCCAAGAATAC-CAAAGTGC
Ppargc1a (Peroxisome proliferator-activated receptor gamma coactivator 1-alpha,PGC-1α)	for<br>rev<br>Probe (FAM, TAMRA)	TCGAAAAAGAAGTCCCATACACAA<br>TTCCACACTTAAGGTTCGCTCAATA<br>CACCAAATGACCCCAAGGGTTCCC
Srebp1c[1] (Sterol regulatory element-binding protein 1c)	for<br>rev	CATCGACTACATCCGCTTCTTG<br>GTGATTTGCTTTTGTGTGCACTTC
Tnfa[1] (Tumor necrosis factor-alpha)	for<br>rev	AGGGATGAGAAGTTCCCAAATG<br>GCTTGTCACTCGAATTTTGAGAAG

[1]Genes were measured by the SYBR-Green method. No probe was needed. Abbreviations: FAM, 6-carboxyfluorescein; TAMRA, tetramethylrhodamine.

4. Supplementary Table S2 (Antibody List, Immunoblots)

Table S2. Blots were probed with the following antibodies.

Primary antibody	Description	Purchased from
phospho-ACC (Ser79)	Monoclonal rabbit anti–phosphorylated ACC, 1:1000	Cell Signaling, Danvers, MA; USA, catalog no. 3661
ACC	monoclonal rabbit anti-total ACC. 1:2000	Cell Signaling, Danvers, MA, USA; catalog no. 3662
phospho-AMPK-α (Thr172)	Monoclonal rabbit anti–phosphorylated AMPKα, 1: 1000	Cell Signaling, Danvers, MA, USA; catalog no. 2535
AMPK-α	monoclonal rabbit anti-total AMPKα, 1:2000	Cell Signaling, Danvers,MA, USA; catalog no. 2603
phospho-AKT (Ser473)	monoclonal rabbit anti-phosphorylated AKT; 1:1000	Cell Signaling, Danvers, MA, USA; catalog no. 4058

AKT	monocloncal rabbit anti-total AKT, 1:2000	Cell Signaling, Danvers, MA, USA; catalog no. 9272
PGC1α	monocloncal rabbit anti-PGC1α, 1:2000	Invitrogen, Carlsbad, CA, USA; catalog. no. PA5-38021
Secondary antibody:		
Anti-rabbit IgG (horseradish peroxidase–linked; Cell Signaling Technology,Danvers, MA, USA catatolg no. 7074) was used as secondary antibody.		

Abbreviations: ACC, acetyl-CoA-carboxlyas; AMPKα, AMP-activated protein kinase alpha subunit; PGC1α, Peroxisome proliferator-activated receptor gamma coactivator 1-alpha, IgG immunoglobulin G.

5. Supplementary Table S3 (Experimental Diets)

Table S3. Information on crude nutrients and energy density of the experimental diets.

		SD	WSD
Name		**complete feed for rats & mice**	**DIO – 45 kJ% fat (Lard)**
Company		Ssniff	Ssniff
Order number		V1534 - R/M-Maintenance	E15744-344
Additional information		complete feed for rats & mice	HF diet for rodents with lard (& soybean oil) correspond to D12451 Research Diets
Metab. Energy	kcal/kg	3225	4615
Metab. Energy	MJ/kg	13.5	19.30
Gross Energy	MJ/kg	16.2	22.50
Fat	kJ%	9	45.00
Protein	kJ%	24	20.00
Carbohydrates	kJ%	67	35.00
Sugar	kJ%	8.8	19.1
Crude Fat	g/kg	33	236
Crude Protein	g/kg	190	220
Crude Fibre	g/kg	50	57
Crude Ash	g/kg	64	53
N free extracts	g/kg	546	400
**Sugar**			
Sucrose	g/kg	-	211.0
Monosaccharides	g/kg	54.0	-
Dissacharides	g/kg		-
Sugar (total)	g/kg	54	211.0
**Carbohydrates**			
Dextrin	g/kg	-	108.0
Polysaccharides	g/kg	359	-
Starch	g/kg	-	68
Carbohydrates (total)	g/kg	413	387
Cellulose powder	g/kg	-	57
Cholesterol	mg/kg	-	-

**Fat Sources**				
soybean oil		%	n/a	2.8
butter fat		%	n/a	-
corn oil		%	n/a	-
pork lard		%	n/a	20.8
palm oil		%	n/a	-
**Fatty Acids**				
butanoic acid	C 4:0	mg/kg	-	-
caproic acid	C 6:0	mg/kg	-	-
caprylic acid	C 8:0	mg/kg	-	-
capric acid	C-10:0	mg/kg	-	-
lauric acid	C-12:0	mg/kg	-	500
myristic acid	C-14:0	mg/kg	0.01	2900
palmitic acid	C-16:0	mg/kg	0.45	53,300
margaric acid	C-17:0	mg/kg	-	-
stearic acid	C-18:0	mg/kg	0.09	29,200
arachidic acid	C-20:0	mg/kg	0.01	70
palmitoleic acid	C-16:1	mg/kg	0.01	6200
oleic acid	C-18:1	mg/kg	0.62	94,200
linoleic acid	C-18:2	mg/kg	1.66	34,600
alpha-linolenic acid	C-18:3	mg/kg	0.23	3700
eicosanoic acid	C-20:1	mg/kg	-	-
eicosadienoic acid	C-20:2	mg/kg	-	-
arachidonic acid	C-20:4	mg/kg	-	-
**Minerals**				
Calcium		g/kg	10.00	92.00
Phosphorus		g/kg	7.00	64.00
Magnesium		g/kg	2.20	23.00
Sodium		g/kg	2.40	20.00
Potassium		g/kg	9.10	97.00
Sulfur		g/kg	n/a	n/a
Chlorine		g/kg	n/a	n/a
Ca/P		%	1.43:1	1.44:1
**Trace Elements**				
Iron		mg/kg	189	168
Manganese		mg/kg	68	95
Zinc		mg/kg	91	65
Copper		mg/kg	15	13
Iodine		mg/kg	2.1	1.2
Molybdenum		mg/kg	-	n/a
Fluorine		mg/kg	-	n/a
Selenium		mg/kg	0.3	0.2
Cobalt		mg/kg	-	n/a
Mineral & trace element mix		%	n/a	6
**Vitamins**				
Vitamin A		I.E./kg	15,000	15,000
Vitamin D3		I.E./kg	1100	1500
Vitamin E		mg/kg	110	150
Menachinone		mg/kg	7	20
Thiamin		mg/kg	18	25

Riboflavin	mg/kg	22	16
Vitamin B6	mg/kg	21	16
Vitamin B12	mg/kg	0,1	30
Nicotinic acid	mg/kg	115	47
Pantothenic acid	mg/kg	40	55
Folate	mg/kg	7	16
Biotin	mg/kg	0.51	300
Choline	mg/kg	1370	920
Vitamin C	mg/kg	not added	n/a
Choline chloride	mg/kg	not added	0,2
Vitamin premix	%	not added	1
**Amino acids**			
Lysine	mg/kg	10,000	18,300
Methionine	mg/kg	3300	7800
Cystine	mg/kg	3500	3900
Met+Cys	mg/kg	6800	11,800
Threonine	mg/kg	7100	9700
Tryptophan	mg/kg	2500	2900
Arginine	mg/kg	11,900	8600
Histidine	mg/kg	4400	6700
Valine	mg/kg	9000	15,400
Isoleucine	mg/kg	7900	12,500
Leucine	mg/kg	13,900	21,900
Phenylalanine	mg/kg	8800	11,400
Phe+Tyr	mg/kg	14,900	23,100
Glycine	mg/kg	8800	4700
Glutamic acid	mg/kg	41,000	49,700
Aspartic acid	mg/kg	17,900	16,400
Proline	mg/kg	12,900	25,300
Serine	mg/kg	9900	13,200
Alanine	mg/kg	8200	6600
**Antioxidant**			
Butylated hydroxytolu-ene = E321	%	n/a	n/a
